# The Exact Nuclear Overhauser Enhancement: Recent Advances

**DOI:** 10.3390/molecules22071176

**Published:** 2017-07-14

**Authors:** Parker J. Nichols, Alexandra Born, Morkos A. Henen, Dean Strotz, Julien Orts, Simon Olsson, Peter Güntert, Celestine N. Chi, Beat Vögeli

**Affiliations:** 1 Department of Biochemistry and Molecular Genetics, University of Colorado Anschutz Medical Campus, 12801 East 17th Avenue, Aurora, CO 80045, USA; parker.nichols@ucdenver.edu (P.J.N.); alexandra.born@ucdenver.edu (A.B.); morkos.henen@ucdenver.edu (M.A.H.); 2 Faculty of Pharmacy, Mansoura University, Mansoura 35516, Egypt; 3 Laboratory of Physical Chemistry, ETH Zürich, ETH-Hönggerberg, Zürich 8093, Switzerland; dean.strotz@phys.chem.ethz.ch (D.S.); julien.orts@phys.chem.ethz.ch (J.O.); guentert@em.uni-frankfurt.de (P.G.); 4 Department of Mathematics and Computer Science, Freie Universität Berlin, Arnimallee 6, Berlin 14195, Germany; solsson@zedat.fu-berlin.de; 5 Institute of Biophysical Chemistry, Center for Biomolecular Magnetic Resonance, Goethe University Frankfurt am Main, Frankfurt am Main 60438, Germany; 6 Graduate School of Science, Tokyo Metropolitan University, Hachioji, Tokyo 192-0397, Japan; 7 Department of Medical Biochemistry and Microbiology, Uppsala University, BMC Box 582, Uppsala SE-75123, Sweden; chi.celestine@imbim.uu.se

**Keywords:** NMR, biological macromolecules, proteins, dynamics, correlated dynamics, exact NOE, structure calculation, structure ensemble, allostery, conformational space

## Abstract

Although often depicted as rigid structures, proteins are highly dynamic systems, whose motions are essential to their functions. Despite this, it is difficult to investigate protein dynamics due to the rapid timescale at which they sample their conformational space, leading most NMR-determined structures to represent only an averaged snapshot of the dynamic picture. While NMR relaxation measurements can help to determine local dynamics, it is difficult to detect translational or concerted motion, and only recently have significant advances been made to make it possible to acquire a more holistic representation of the dynamics and structural landscapes of proteins. Here, we briefly revisit our most recent progress in the theory and use of exact nuclear Overhauser enhancements (eNOEs) for the calculation of structural ensembles that describe their conformational space. New developments are primarily targeted at increasing the number and improving the quality of extracted eNOE distance restraints, such that the multi-state structure calculation can be applied to proteins of higher molecular weights. We then review the implications of the exact NOE to the protein dynamics and function of cyclophilin A and the WW domain of Pin1, and finally discuss our current research and future directions.

## 1. Introduction

Proteins exist in equilibrium between many structural states, and as such, are inherently dynamic systems. They sample conformational states that cover large amplitudes and timescale ranges, spanning 10^−11^ to 10^−6^ m and 10^−12^ to 10^5^ s, respectively. While most proteins have been represented as single state-structures in the past, it is becoming increasingly apparent that dynamics are important to their functions [1,2,3,4]. In parallel, it is recognized that concerted motions may be a pivotal factor in the enzymatic function of proteins and in protein–ligand interactions [5,6,7,8]; examples include concerted motions and allosteric responses upon ligand/substrate binding or dissociation [1,2,3,5,9], as well as changes in protein volume, which are correlated with internal motion [4]. Spatial sampling, entropic changes, and allosteric communication within structured and disordered regions have also been shown to influence enzyme catalysis and allow for adaptive responses [5,6,8]. In addition, dynamics are integral to intrinsically disordered proteins, which lack clear-cut energy minima, often form ternary complexes and can have many binding partners [7]. Another prime example is the protein folding process, which comprises dynamics of a complex nature on large scales in both time and space. Currently, the most common way to investigate protein folding is through molecular dynamics simulations [10,11], and through thermodynamic and kinetic studies [8,12,13]. For a detailed understanding of protein function at the atomic level, not only are 3D atomic-resolution structures a prerequisite, but an accurate description of their dynamic properties is also required [10,14,15]. NMR spin relaxation methods have long been used to characterize these properties [16]—yet the analysis of such data beyond single-site flexibility and exchange, such as allosteric motions, relies heavily on molecular dynamic simulations [16,17] or statistical model selection techniques [18].

One of the major challenges in structural biology is thus a comprehensive description of the 3D structures and the exchange dynamics between structural states at the atomic resolution. Conventional NMR structure determination relies heavily on nuclear Overhauser enhancement (NOE) rate constants to determine an averaged conformation of the protein structure. However, this method has been described as representing “the shape of the molecule as it would be seen on a photograph taken at low shutter speeds” [19], and furthermore, “the average derived from spectroscopic data represents a virtual structure devoid of physical meaning” [19]. While this view is over-pessimistic, it does reflect the fact that an NMR observable is an averaged property rather than an exact representation. For example, if a single residue on a protein fluctuates in its distance relative to a nearby residue, the NOE measured between atoms of those two residues is an average between the two states, leading to a calculated distance that lies between the two exact distances. The well-established standard NMR structure calculation protocol makes use of these overabundant and experimentally readily accessible NOE rate constants—typically up to 20 per residue for small proteins [20]. Although the NOE rate constant is proportional to the inverse 6th power of the distance between two dipolar interacting spins (*r*^−6^) [21], these rate constants are employed in a semi-quantitative manner at most because the measurement of NOEs is compromised by various interfering mechanisms throughout the pulse sequence [22], and also by spin diffusion [23]. Thus, the calculated structures determined from conventional NOEs fail to represent the dynamic nature and exact structure of the proteins by which they are measured, and the protocol for the generation of atomic-resolution spatial representations still needs improvement, due to the difficulty in detecting translational and concerted motions.

**Figure 1:**
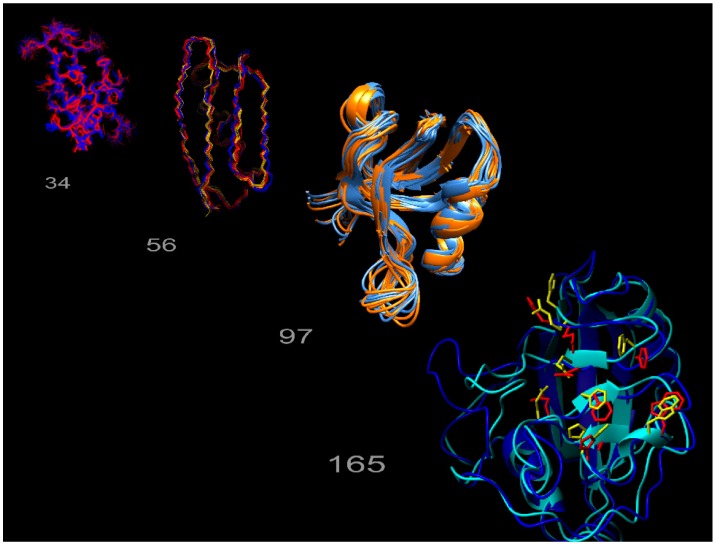
Exact nuclear Overhauser enhancement (eNOE) structure gallery. The multi-state ensembles calculated from eNOEs for the WW domain of Pin1 (34 residues), the third domain of immunoglobulin binding protein G (GB3; 56 residues), the second post-synaptic density-95/discs large/zonula occludens-1 (PDZ2) domain from human tyrosine phosphatase 1E (97 residues), and human cyclophilin A (165 residues), are shown from left to right.

We previously developed an ensemble-based structure determination protocol using ensemble-averaged distance restraints obtained from exact NOE (eNOE) rate constants, and applied it to the model protein GB3 (third immunoglobulin binding domain of protein G) [24,25]. This work showed that eNOEs could be used to obtain multi-state structural ensembles of GB3 that described the experimental data better than the single-state ensemble. These multiple states allowed for the concerted motion across a β-sheet and interconversion between α-helix states of GB3 to be represented. The exceptional definition of the ensemble is due to the high precision and accuracy of the eNOEs [26]. For example, we showed that the measurement of eNOEs [27,28] between amide protons in perdeuterated and protonated human ubiquitin and GB3 enabled the determination of distances up to 5 Å with less than a 0.1 Å error [29,30]. The nature of eNOEs results in extremely tight structural bundles with very low root-mean-square deviations (RMSDs) when compared to conventional structure calculations. Interestingly, using eNOEs for single-state structure calculations results in many distance restraint violations, which are indicative of the structure not agreeing well with the experimental data. We found that these violations can be attributed to the motion-averaged nature of the measured eNOEs, which carry information about the spatial dynamics of mobile atoms in a protein. The distance restraints can be satisfied by allowing the structure calculation to assume multiple states, thereby capturing dynamics information regarding the protein or biomolecule of interest. This is exemplified in Figure 1, which depicts the multi-state ensembles we have calculated so far. The gallery consists of those for the WW domain of human Pin1, the third domain of immunoglobulin binding protein G (GB3), the second post-synaptic density-95/discs large/zonula occludens-1 (PDZ2) domain from human tyrosine phosphatase 1E, and human cyclophilin A. Thus, eNOEs lend unprecedented precision to the calculation of distance restraints used for structure calculation.

We have recently investigated the experimental accuracy of uni-directional eNOEs [22], and have extended the eNOE data set of large proteins by the evaluation of NOEs with unresolved diagonals [31]. We have also extended our previously reported eNOE data set for the protein GB3 by a very large set of backbone and side-chain residual dipolar couplings (RDCs) and three-bond *J* couplings [32], and have investigated how much structural and dynamics information is shared between the eNOEs and other NMR probes [33]. We have demonstrated that at least four structural states are required to represent the complete data set for GB3 by dissecting the contributions to the CYANA target function (TF) [34]. Finally, we have applied our eNOE-based multi-state ensemble protocol to the regulatory WW domain of the human mitotic regulator Pin1 [35], as well as another human prolyl isomerase, cyclophilin A [36]. These recent advances will be discussed in Section 2, after a short general overview of the eNOE methodology in the remainder of Section 1.

### 1.1. The Exact Nuclear Overhauser Enhancement Extraction Protocol

**Figure 2:**
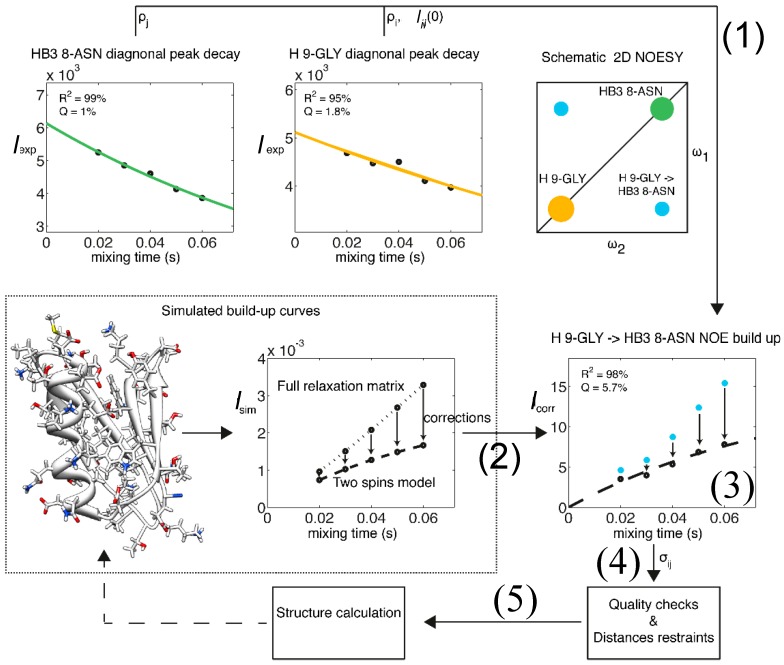
Flow chart representation of the eNORA structure determination protocol using eNOEs. As an example, the eNOE originating from the amide H of Gly9 (spin *i*, orange) and enhancing H^β3^ of Asn8 (spin *j*, green) of GB3 is shown. (1) The diagonal-peak intensities derived from the NOESY spectra are fitted to mono-exponential decay functions to extract the auto-relaxation rate constants *ρ_i_* and *ρ_j_*, and the initial magnetizations, Δ*M_ii_*(0) and Δ*M_jj_*(0); (2) A build-up curve taking into account all magnetization pathways is simulated with the full relaxation matrix approach. This simulation requires a 3D structure as input, which may be based on a conventionally determined structure with sufficient accuracy; (3) Corrections for the intensities at each mixing time are applied to the experimental NOE build-ups; (4) The NOE build-up intensity is fitted, the quality of the fit is evaluated, and upper- and lower-bound distance restraints are created; (5) A structure calculation is performed with the new distance restraints using established packages such as CYANA [39,40]. This structure may be used as an input for (2) in a new cycle, as indicated by the broken arrow. Adapted from [38] with permission from ACS, Copyright (2012).

It is still routine in protein structure determination to employ NOEs as upper distance restraints rather than exact average values [20]. This practice has resulted from the difficulty in converting NOEs into exact distances for large biomolecules [27]. However, with the introduction of the 3D nuclear Overhauser effect spectroscopy (NOESY) and augmented spectrometer sensitivity, it is now possible to convert NOE build-ups into exact distance restraints. We established a protocol to extract eNOEs between two spins, *i* and *j*, using an optimized 3D heavy atom-resolved [1H,1H]-NOESY experiment, ideally on cryogenic probes [26]. Extraction of the corresponding cross-relaxation rate is severely hampered by spin diffusion that is relayed via neighboring spins [23,37]. Therefore, we developed two approaches for the extraction of the rate that take into account the error obtained from spin diffusion [29,30]. These protocols are encoded into the MATLAB program eNORA (exact NOE by relaxation matrix analysis) [38] and its successor, eNORA2 (see below). The eNORA routine fits the NOESY-derived diagonal-peak intensities to mono-exponential decay functions to extract the auto-relaxation constants, *p_i_* and *p_j_*, and the initial magnetizations, Δ*M_ii_*(0) and Δ*M_jj_*(0). In the first approach, cross-peak build-up curves are then simulated with the full relaxation matrix approach [23] applied to a conventionally determined NMR structure or an X-ray structure. This approach corrects for spin diffusion because the magnetization transfer pathways between all spins are active simultaneously. Corrections for the intensities at each mixing time are derived from the simulation and applied to the experimental intensities. The corrected cross-peak build-up curves are fitted by using *p_i_*, *p_j_*, Δ*M_ii_*(0), and Δ*M_jj_*(0) as fixed input parameters, and the cross-relaxation rate constants *σ_ij_* and *σ_ji_* as free variables. The quality of the fit is evaluated, and *σ_ij_* and *σ_ji_* are converted into distance restraints *r* through the relationship *σ* ∝ *r*^−6^. A structure calculation is then performed with the new distance restraints using software packages such as CYANA [39,40]. The newly calculated structure may then be used as an input for the next correction simulation and for refinement of the structure. A schematic representation of our eNORA protocol is shown in Figure 2.

The second approach, originally coded in our diffusion of magnetization in NOESY (DOMINO) script [30], accounts for spin diffusion by summing individual correction contributions from each neighboring spin *k* obtained from the exact solution of the expected peak intensity modulations of three-spin systems *ijk*. This approach is well-suited for partially deuterated proteins [38]. We have shown that this approach is in agreement with the full relaxation matrix protocol for a large range of molecular overall tumbling and NOESY mixing times where the spin diffusion is easily traceable [38].

### 1.2. Exact Nuclear Overhauser Enhancements to Determine Dynamics through Multiple-State Ensembles

**Figure 3:**
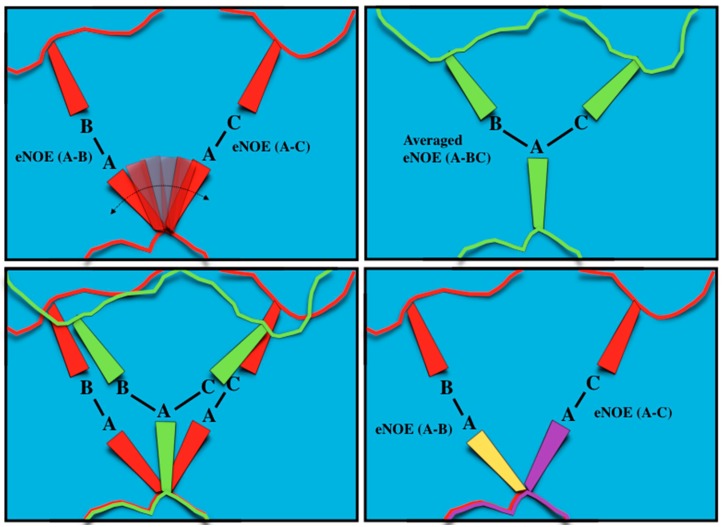
Schematic representation of single-state vs multiple-state eNOE structure calculation. (**Top, left**) A theoretical system of atoms A, B, and C within a protein. Atoms B and C are fixed at certain positions, while atom A exists in dynamic equilibrium, where it is in close proximity with both atom B and atom C; eNOEs would be measured between both atoms A and B as well as atoms A and C; (**Top, right**) The eNOEs measured appear to be from atom A interacting with atoms B and C at the same time. Assuming a single-state structure causes atom A to be placed directly between atoms B and C, and causes atoms B and C to be brought closer to atom A, distorting the calculated structure away from the true positions; (**Bottom, left**) Representation of how atoms A, B, and C in the calculated single-state structure (green) are distorted from their real positions (red); (**Bottom, right**) Allowing the structure calculation to employ multiple states (yellow and purple) allows atom A to occupy its true positions between atoms B and C, thus satisfying the eNOE-derived distance restraints and capturing important dynamics information.

To understand how allowing for multiple states in structure calculations alleviates the distance restraint disagreements associated with single-state calculations, and why multiple states can represent the dynamics of the protein, a simple three-atom example may be considered, as shown in Figure 3. In this theoretical protein, atoms B and C are fixed at determined positions, while atom A exists in a dynamic equilibrium where, half of the time, it is in close proximity to B, and the other half, it is in close proximity to C (top, left). For this system, eNOEs would be measured between atoms A and B and between atoms A and C, and would then be used as inputs for the structure calculation. While the eNOEs measured between atom A and atoms B and C are due to two separate states, the eNOE appears to be due to atom A interacting with atoms B and C at the same time, leading the averaged eNOEs to represent an apparent distance that is longer than when atom A is closest to atoms B and C (top, right). This averaging appears in the cross-relaxation rate as *σ* ∝ *r*^−6^, and is thus extremely sensitive to the distance between the atoms. Therefore, the cross-relaxation rate is dominated by the points in time when atom A is closest to atoms B and C, splitting the eNOE into two main signals representing those two states. If a single-state structural ensemble is then calculated with these averaged eNOEs, atom C becomes positioned between its two true positions and the positions of atoms A and B are brought towards atom C in order to reduce the distance restraint violations, which are strongly enhanced by the nonlinearity of the distances’ dependence on the NOE (top right, bottom left). The shortcoming of a single-state model is obvious, especially when we scale this scenario up to the size of an entire protein, leading to a distorted and unphysical structural representation. If the structure calculation process is instead allowed to satisfy its distance restraints by assuming multiple states, atom A is allowed to exist in two states, in proximity to either atom B or atom C, better satisfying its distance restraints and capturing true dynamic information about the atom (bottom, right). This principle may be generalized to more than two states, however, we use the minimal number of states in order to prevent an over-fitting of the data. Thus, in contrast to the standard structure determination protocol, eNOEs take into account the fact that the NOE is a time- and ensemble-averaged parameter.

We note that our treatment of time-averaging is strictly correct for motion on timescales slower than nanoseconds. Although not implemented at this point, motion that is even slower than the chemical shift timescale (slower than ca. 100 ms), resulting in peak splitting, could easily be accounted for by extracting separate distances from the split-peak components. Fast motion (faster than nanoseconds), on the other hand, which typically exhibits smaller amplitudes than slow motion, is treated in an approximate manner [41].

### 1.3. Definitions of States, Conformers and Ensembles

**Figure 4:**
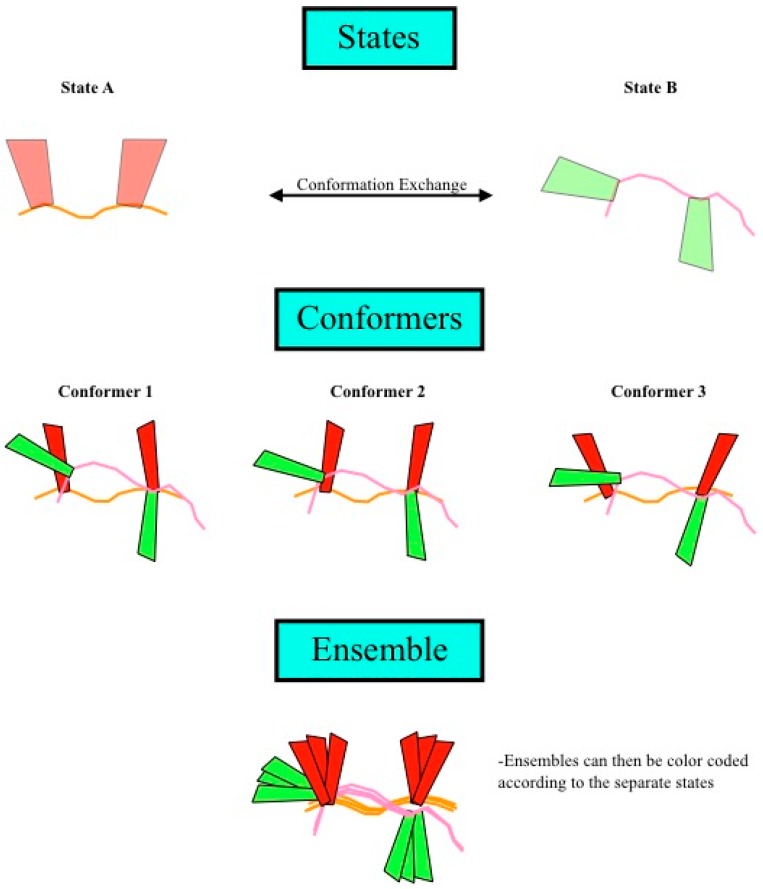
Definition of states, conformers, and ensembles. (**Top**) Shown are two different states of a theoretical protein, with each individual state being defined as one set of coordinates for all the atoms of a molecule. Structure calculation using this data would result in a single conformer that satisfied the data well and could be composed of one or more states; (**Middle**) The structure calculation is repeated multiple times, resulting in many conformers; (**Bottom**) Finally, the ensemble is the superposition of all of the conformers. If multiple states were allowed in the structure calculation, then the ensemble could be divided into sub-bundles, each of which would be identified by structural similarity in the region of interest, which corresponds to a single state.

Before we go further, it is useful to define many of the terms that we have been using in the text. A structure is defined by a bundle (or an ensemble) of conformers fulfilling the experimental data. A conformer is the result of one individual structure calculation that fulfills the experimental data and may be composed of one or more states. A state is one set of coordinates for all atoms of a molecule. If there are multiple states, they fulfill the experimental data on average and not individually. Sub-bundles are formed by sorting the states contained in a set of conformers according to structural similarity in the region of interest. There are as many sub-bundles as there are states in a conformer, and each sub-bundle comprises as many conformers as the original structure bundle. This requires each state to belong to exactly one sub-bundle. The sub-bundle for each structural state is a measure of the precision of the individual structural state, similar to the conventional bundle representation. This description is represented graphically in Figure 4. It is important to note that these states do not necessarily correspond to states given by energy minima, instead, eNOEs only have the potential to resolve these states.

## 2. Exact Nuclear Overhauser Enhancement Recent Advances

### 2.1. Exact Nuclear Overhauser Enhancement Methodology

**Figure 5:**
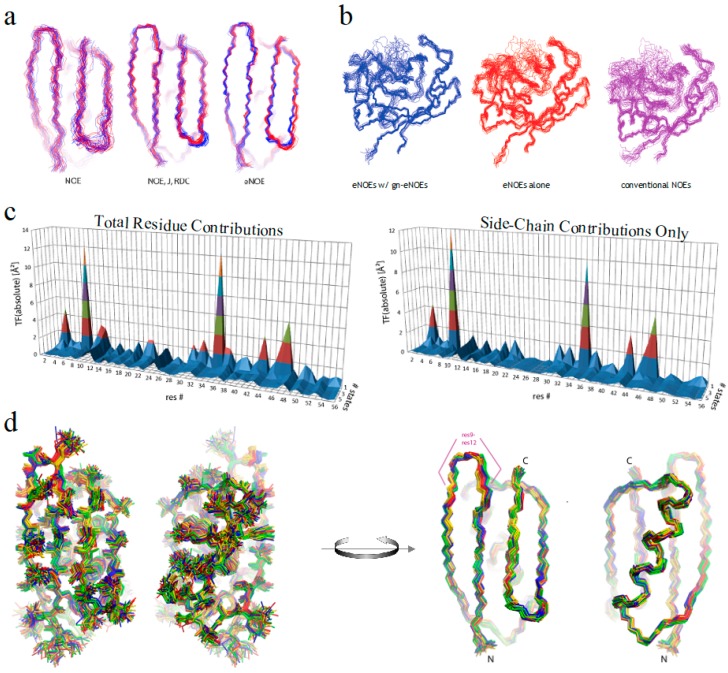
Recent eNOE advances. (**a**) Two-state ensembles of GB3 calculated from conventional NOEs, NOEs supplemented with RDCs and *J* couplings, or from eNOEs alone, from right to left, respectively. Adapted from Journal of Structure Biology, 191, Vögeli, Olsson, Riek, Güntert, Complementarity and congruence between exact NOEs and traditional NMR probes for spatial decoding of protein dynamics [33], 306–317, Copyright (2015), with permission from Elsevier; (**b**) Single-state structural ensembles of cyclophilin A calculated using 1254 eNOEs supplemented with 2217 generic normalized eNOEs (left), 1254 eNOEs alone (middle), or using 4537 conventional NOEs (right). Adapted from [31], Copyright (2015), with permission from Springer; (**c**) Residue-specific target function (TF) values. The individual contributions are plotted versus the residue number and the number of states on the *x* and *y* axes, respectively. On the left are the contributions from the entire residue, while the right are only those from the side-chain atoms; (**d**) Heavy-atom (left) and backbone (right) representations of the four-state GB3 structural ensemble are shown. The 20 conformers with the lowest TF values were selected, and the four states colored gold, red, green, and blue were obtained by grouping the loop comprising residues 9–12. Images (**c**) and (**d**) were both reprinted from [34].

We have recently shown that eNOE distance restraints contain more information than those derived from conventional NOEs, and eNOE data alone offered as much information as conventional NOE data in combination with abundant RDC and *J* coupling data [33]. Figure 5a shows multi-state ensembles of GB3 calculated with conventional NOEs alone, supplemented with *J* couplings and RDCs, or with just eNOEs. This finding is significant, as the experimental effort required to obtain eNOEs is significantly less than to collect *J* couplings and RDCs. Using this data set encompassing eNOEs, RDCs and *J* couplings, which is significantly larger than that used in our previous ensemble calculation [24,25], we dissected residue-specific contributions from GB3 to the CYANA TF [34]. Figure 5c left shows that the major contributions to the TF were from residues 8 and 35, which underwent drastic decreases in their TF upon an increase in the number of states. We performed the same analysis with just the side-chain atoms in Figure 5c right, and found that it mimicked the outcome of the total-residue analysis, again, with a significant decrease upon the use of additional states. This indicated that the largest decrease to the TF comes from allowing side chains to populate additional χ^1^ rotamer states, further supporting the fact that eNOEs can capture important dynamics information. In contrast to our previously published three-state ensemble of GB3, we found that the optimal number of states to accurately represent the dynamics of GB3 was four. The four-state structural ensemble of GB3 is shown in Figure 5d, which was very compact, and well defined by the input data set.

In addition, we established a protocol to obtain accurate distance restraints from uni-directional eNOEs (NOEs for which only one of the symmetry-related cross peaks can be evaluated) and from NOEs with unresolved diagonals, significantly increasing the number of distance restraints that can be extracted, and thus being used in the structure calculation [22,31]. Through this analysis, we justified our previously published choice of additional tolerances to upper and lower distance limits of ±15% to ±20% for uni-directional eNOEs. Importantly, we found that, as opposed to our common practice, normalization of the cross-peak intensities to the diagonal-peak intensity of the spin of the destination magnetization rather than the origin of magnetization works equally well for 2D NOESY, and even better for 3D NOESY-HXQCs (where the HXQC stands for HSQC or HMQC). This finding opens up the possibility to collect and use many more uni-directional eNOEs in the structure calculation. For NOEs with unresolved diagonals, we introduced a protocol to increase the number of eNOEs that can be obtained from proteins of larger size, while avoiding adding semi-quantitative restraints, such as those obtained from conventional NOEs, that may induce distance errors into the structure. We normalize such eNOE buildups to a value that is chosen to be larger than any fitted diagonal-peak height of the entire molecule. We have termed these as generic normalized eNOEs (gn-eNOEs). This data does not enforce an incorrect separation of states, thus allowing eNOE-based multi-state ensemble calculations to be applied to larger, biologically active proteins. Figure 5b shows the impact of supplementing eNOEs with gn-eNOEs on the 165-residue enzyme cyclophilin A, which resulted in a much tighter bundle than with eNOEs alone or with conventional NOEs. We have also developed a method for stereospecific assignments for the majority of relevant diastereotopic groups by comparing eNOE-derived distances to protein structure bundles calculated without stereospecific assignments, making it possible to obtain more detailed structural and dynamical information from NOEs [42].

### 2.2. The eNORA2 Program

**Figure 6:**
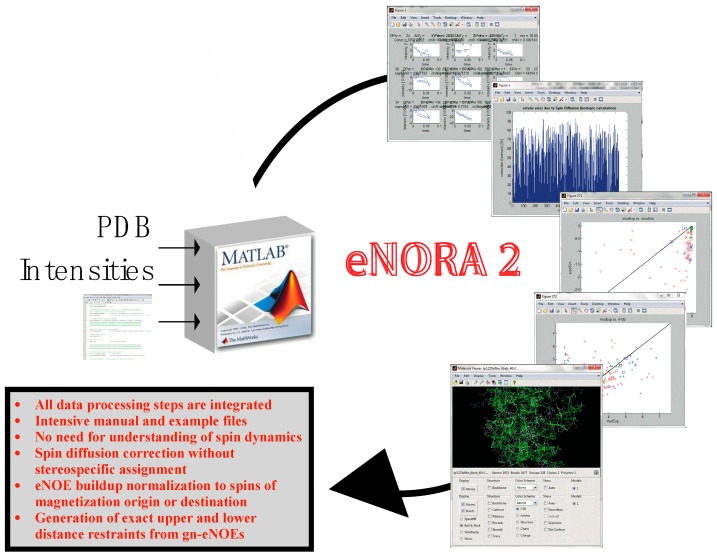
The eNORA2 program for eNOE analysis; eNORA2 significantly speeds up and automates the process of eNOE extraction and data processing, decreasing the time required from several months to ca. 2 weeks. See reference [43] for more information regarding this program.

In our initial studies, NOE buildup fitting, spin-diffusion correction, book keeping, and upper and lower distance limit generation involved intensive manual work and thus, was very time consuming. The eNOE analysis also required a deep understanding of the underlying principles. Therefore, we have developed an extensive MatLab package for eNOE analysis, named eNORA2 (see Figure 6) [43] that supersedes the previously published eNORA [38] and DOMINO programs [30]. The package is written for experts and non-experts alike, and it speeds up the process considerably. No understanding of spin dynamics is required any longer. The program integrates all data processing steps required to convert intensities of assigned peaks in NOESY series into upper and lower distance limits for structure calculation. Notable improvements over the original version of eNORA include options to correct for spin diffusion without stereospecific assignment, saving plots of fits to cross-peak and diagonal-peak intensities as separate files, and the addition of mixing times. There are also options for buildup normalization to diagonal-peak intensities of spins of magnetization origin or destination, the generation of upper and lower distance files for structure calculation with the CYANA package, and the generation of upper limits from gn-eNOEs. We provide an extensive user manual and example files.

### 2.3. Exact Nuclear Overhauser Enhancement Analysis of Pin1 WW domain

**Figure 7:**
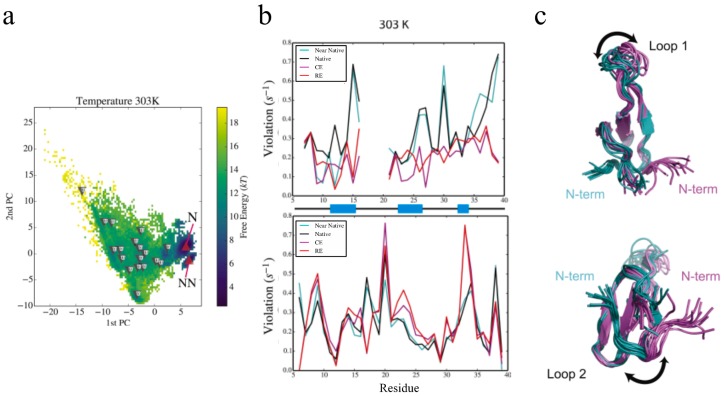
The eNOE is sensitive to minor populated states in the Pin1 WW domain. (**a**) Free-energy landscapes and cluster populations from replica-exchange molecular dynamics (MD) simulations and maximum entropy-based chemical shift reweighing carried out at 303 K are shown. The upward facing red triangles indicate cluster centroids that have a higher population in the reweighed ensemble (RE) than the canonical ensemble. The downward pointing triangles indicate clusters that have a lower population. For more information, see reference [35]; (**b**) Root-mean-square deviation (RMSD) violations of cross-relaxation rate constants from bidirectional (top) and unidirectional (bottom) eNOEs from REs and canonical ensembles (CEs), as well as from the native and near-native conformational clusters at 303 K. RMSD violations of 0.1–0.2 s^—1^ translate into distance errors of approximately 0.3–0.4 Å. Secondary structure by residue (black lines indicate loops and coiled regions, and blue blocks indicate β strands) are shown on *x*-axis; (**c**) The conformational changes inherent to the native and near-native states are depicted by calculated structural ensembles. The change in loop 1 (residues 17–20, top) of the WW domain and the topological rearrangement of the N and C termini (bottom) are especially prevalent. Ten conformers are shown in each cluster. This figure was adapted from [35], Copyright (2016), with permission from Cell Press.

The advances lined out above have allowed us to extend our eNOE protocol to an assortment of proteins, as shown before in Figure 1. For one of the proteins in the gallery, the 34-residue peptidyl-prolyl *cis-trans* isomerase Pin1-WW, we have been able to elucidate information regarding allosteric signal transduction in the WW domain upon ligand binding [35]. Pin1 contains two domains, a flexible N-terminal binding domain (WW), which is tethered via a flexible linker to a larger C-terminal domain that contains the peptidyl-prolyl *cis-trans* isomerase (PPIase) activity [44,45]. Both domains interact with a wide range of ligands containing phosphorylated Ser/Thr-Pro motifs [44]. Interestingly, it has been shown that there is significant cross-talk between the two domains by means of loop 2 of the WW domain (residues 27–30) and residues 138 and 140–142 in the PPIase domain [46,47,48,49]. For example, a binding event in one domain alters the binding affinity in the other domain. The allosteric communication is mediated by dynamic circuits, as demonstrated by extensive methyl and backbone NMR relaxation measurements [49,50]. The spatial aspects of the dynamic nature of Pin1 have been difficult to capture experimentally, but with the help of eNOEs, a comprehensive picture of its dynamics is coming into view. Ensembles of Pin1 generated using a combination of replica-exchange molecular dynamics (MD) simulations and maximum entropy-based chemical shift reweighing [51,52] resulted in two highly probable states, termed the native and near-native states, and many less-defined unfolded states, shown in Figure 7a. Our eNOEs recorded from the WW domain alone were highly sensitive to the presence of the near-native state and also to the unfolded states, as shown by the RMSD violations in Figure 7b, which was remarkable considering that the near-native state was lowly populated (~5–10%). This analysis suggested that Pin1 exists in its native conformation shown in Figure 7c (magenta), and an energetically excited near-native state conformation shown in Figure 7c (teal). One of the major characteristics of the near-native state was a topological rearrangement of the N- and C-termini, as well as in loop 1 (residues 17–20, Figure 7c, top) of the WW domain. In addition, there was an altered preference of the backbone dihedral angles in the binding loop. In the native state, the WW domain of Pin1 might be free to interact with the PPIase domain, but this interaction may be obstructed in the near-native state. The equilibrium between these two states might be allosterically modulated by the binding of different ligands, thus possibly allowing for the recruitment of Pin1 for a variety of different functions. Further analysis by eNOEs might be able to determine the spatial sampling of not only the WW domain, but the entire Pin1 protein. This is a current goal of our lab and is discussed in Section 3.

### 2.4. Exact Nuclear Overhauser Enhancement Analysis of Cyclophilin A

**Figure 8:**
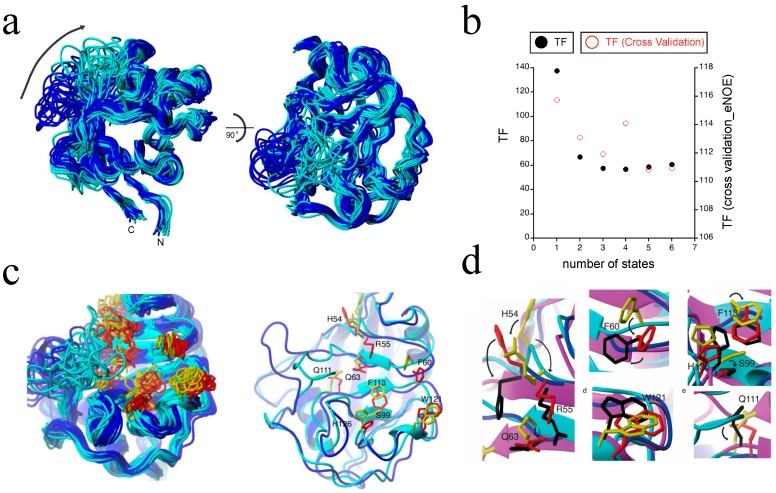
The eNOE detection of an allosteric network in cyclophilin A. (**a**) A structural ensemble of cyclophilin A in its apo form is shown, highlighting the presence of two distinct states. Each of the 20 conformers represents two states. States were color-coded as open (blue) and closed (cyan). Two distinct states are observed throughout most of the structure. The orientation of the structural ensemble shown on the right is a 90° rotation of that on the left; (**b**) The dependence of the CYANA TF (black) and the overall TF from the jackknife-type cross-validation (red) are shown as functions of the number of states; (**c**) The two-state ensemble of the active site residues of cyclophilin A. On the left, a ribbon representation of the 20 conformers is shown, color-coded individually for the two states: the closed state is shown in cyan for the backbone and in yellow for the side chains of the active site, while the open state is shown in blue for the backbone and in red for the side chains in the active site. The right shows a single representative from each state, with the side chains labeled. The lowest energy two-state conformers were selected; (**d**) Proposed mechanism of action of cyclophilin A at the atomic resolution. The X-ray structure of cyclophilin A in complex with the HIV-1 capsid protein (PDB ID: 1ak4 [59]) was superimposed with the presented two-state ensemble, which highlights the fact that the open-state matched the ligand-bound state well. The closed state is shown in cyan for the backbone ribbon and in yellow for the side chains, the open state in blue for the backbone ribbon and in red for the side chains, and the X-ray structure is shown in purple for the backbone ribbon and in black for the side chains. Individual close-ups of the superposition are shown. The potential modes of action for catalysis of the individual residues are indicated by arrows. Reprinted from [36], Copyright (2015), with permission from Wiley.

The largest protein to which we applied our eNOE-based multi-state ensemble protocol to date is the well-studied 165 residue peptidyl prolyl *cis-trans* isomerase cyclophilin A [53,54,55,56]. Previous studies suggested that the mechanism of action involves a dynamic network between the enzyme’s active site and nearby segments, and involves an electrostatic handle mechanism at the carbonyl group of the residue proceeding the proline in the substrate [56,57,58]. For such systems, eNOEs can significantly contribute to the elucidation of the dynamics. In order to obtain an experimentally derived description of the various substates of cyclophilin A at atomic resolution, we calculated multi-state ensembles using eNOEs and RDCs [36]. The use of the gn-eNOEs was crucial in this study because there were not enough bi-directional eNOEs present to resolve the dynamic loop of the enzyme. Supplementing our data set with gn-eNOEs, however, doubled the number of eNOEs used in the bundle calculation [31]. As can be seen in Figure 8b, the CYANA TF (black points) decreased as the number of states was increased from one to three, indicating that multiple states were necessary to describe the eNOE data well. These findings were cross-validated via a jackknife procedure (red points) as well as with RDCs and ^3^*J*_HNHa_ couplings [36], which again all decreased with increasing the number of states. For structural analysis, we chose 20 two-state conformers, as shown in Figure 8a, in order to avoid over-fitting the data. The ligand binding loop comprising residues 64–74 sampled two spatially well-separated states. We termed these two states as the “open” state (blue) and the “closed” state (cyan) because the closed state was slightly more compact. The two states were also distinct in their active sites and in the surrounding regions, indicating long-range correlations. In addition, our two-state ensemble was able to capture the dynamic profile of the side chains seen within the active site of the enzyme, shown in Figure 7d and Figure 8c. Importantly, we found that these sampled states resolved the proposed activity-related dynamic network at an atomic resolution [56], which guided the charged side chain of R55 into position to create an electrostatic potential that acted on the carbonyl group of the proline-preceding residue of the ligand [58]. In addition, the side chains of the open state (blue for backbone, red for side chains) and closed state (cyan for backbone, yellow for side chains) closely matched those of the crystal structure of cyclophilin A in complex with the HIV-1 capsid protein (purple for backbone, black for side chains; PDB ID: 1ak4) [59], as shown in Figure 8d. Thus, we were able to determine a two-state model of cyclophilin A in the apo state, which revealed a long-range and well-orchestrated conformational interchange between sub-states important for its catalytic activity. This highlighted a synergistic induced-fit and conformational sampling mechanism of action, and further showed the validity of eNOE-calculated multi-state ensembles for the determination of protein dynamics.

## 3. Current Research and Development

### 3.1. Applying Exact Nuclear Overhauser Enhancements to Pin1

As mentioned before, we previously used our eNOE protocol to investigate the dynamics of the Pin1-WW domain, which revealed dynamics information that potentially explained how the WW domain influences the mechanism of action of Pin1. However, these results were somewhat speculative, as they only involved the WW domain of the protein. We are currently extending our eNOE approach to the entire Pin1 protein. Multi-state ensembles may provide an unprecedented spatial representation of the allosteric mechanism between the two domains at the atomic resolution.

### 3.2. Extension of Exact Nuclear Overhauser Enhancements to the Proteasome

**Figure 9:**
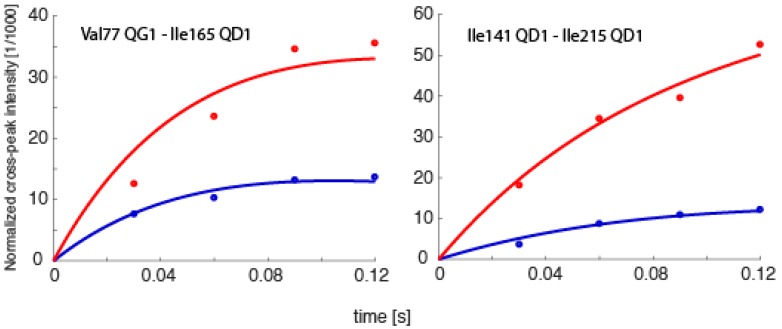
NOE buildup curves obtained from the 360 kDa half-proteasome. Bi-directional buildup intensities for the spin pairs Val77Qγ1–Ile165Qδ1 and Ile141Qδ1–Ile215Qδ1 are shown. The intensities are normalized to the diagonal-peak intensities at the onset of mixing, which are obtained together with auto-relaxation rates from fits to the diagonals.

Spin relaxation, the same phenomenon that allows for NOEs to be measured, also ultimately defines the upper size limit of molecules that NMR can be applied to. Generally, proteins larger than ~35 kDa have such fast transverse relaxation rates (*T*_2_ relaxation times) that the peaks are too broad to obtain any data from. However, there are exceptions. Recently, spin relaxation measurements were obtained from methyl groups in various deuterated constructs of the 20S proteasome of *Thermoplasma acidophilium*, some as large as 1.1 MDa, which directly allowed for the extraction of order parameters characterizing angular motion amplitudes on the sub-nanosecond timescale, the extraction of RDCs containing orientational information, or studies involving paramagnetic samples [60,61,62]. While exciting, these probes were all complementary to the most significant form of structural/dynamics information, internuclear distances [33]. Therefore, we are currently testing whether it is possible to determine exact time-averaged distances using eNOEs between methyl groups in the 360 kDa half proteasome from *Thermoplasma acidophilium*. Preliminary results demonstrate the feasibility of obtaining uni- and bi-directional NOE buildups, as exemplified in Figure 9. We anticipate that our work will open up an avenue for eNOE measurements on molecules of at least one megadalton weight. One significant application of such measurements would be the detection of relative changes in proton–proton distances upon induction of structural changes by ligand binding.

### 3.3. Applying F_1_F_2_-Selective NMR Spectroscopy to Exact Nuclear Overhauser Enhancements

Recently, it has been shown that insertion of the solution-state Hartmann–Hahn cross-polarization (CP) [63,64] element prior to 2D pulse-sequences gives the ability to reveal information equivalent to that of conventional 4D experiments [65]. The selectivity of CP means that the resulting spectra are free of overlap common to higher-dimensional NMR spectra and are easy to assign, and measurement times are drastically reduced if only certain nuclei are of interest [65]. This technique is particularly attractive for our eNOE protocol, as the *F*_1_*F*_2_-selective CP element can be inserted into the conventional NOESY experiment to determine NOE buildups at varying mixing times in a fraction of the typically long time required to measure a series of complete 3D or 4D NOESY spectra. The reduced measurement time and peak overlap would allow us to more easily extend eNOEs to track structural changes induced by ligand binding or by allosteric effects in larger proteins. Indeed, *F*_1_*F*_2_-selective NOESY experiments could find significant use in the abovementioned studies of the 20S proteasome from *Thermoplasma acidophilium*.

## 4. Conclusions

In conclusion, we have presented the latest advances in the eNOE methodology and its application to biological systems. More original aspects of the eNOE and multi-state structure calculation have been reviewed in previous publications [26,66,67]. We believe that the eNOE technology is a highly versatile tool that will help with answering diverse questions in structural biology.
